# Impacts of environmental factors on the climbing behaviors of herbaceous stem‐twiners

**DOI:** 10.1002/ece3.3479

**Published:** 2017-10-08

**Authors:** Liang Hu, Youfang Chen, Meicun Liu

**Affiliations:** ^1^ Geography and Planning School Sun Yat‐sen University Guangzhou China

**Keywords:** environmental factors, external support, illumination, inclination, plant development and life history traits, temperature, twining curvature

## Abstract

The curvature of the helical trajectory formed by herbaceous stem‐twiners has been hypothesized to be constant on uniformly sized cylindrical supports and remains constant on different supports varying in diameter. However, experimental studies on the constant curvature hypothesis have been very limited. Here, we tested the hypothesis in a series of experiments on five herbaceous stem‐twiners (*Ipomoea triloba*,* Ipomoea nil*,* Phaseolus vulgaris*,* Vigna unguiculata,* and *Mikania micrantha*). We investigated how internode characteristics (curvature [β], diameter [*d*], and length [*L*]) and success rate (SR) of twining shoots would be affected by support thickness (*D*), temperature (*T*), illumination, and support inclination. The results showed that: (1) the SR of tested species decreased, but *d* increased with increasing support thickness. The β of the twining shoots on erect cylindrical poles was not constant, but it decreased with increasing *d* or support thickness. (2) The SR of tested species was not obviously reduced under low‐temperature conditions, but their β was significantly higher and *d* significantly lower when temperature was more than 5°C lower. (3) The SR *, d,* and *L* of two tested *Ipomoea* species significantly declined, but β increased under 50% shading stress. (4) The curvatures of upper semicycles of *I. triloba* shoots on 45° inclined supports were not significantly different from curvatures of those shoots climb on erect supports, whereas the curvatures of lower semicycles were 40%–72% higher than curvatures of upper semicycles. *Synthesis*: Our study illustrates that stem curvatures of a certain herbaceous stem‐twiners are not constant, but rather vary in response to external support, temperature, and illumination conditions. We speculate that herbaceous stem‐twiners positively adapt to wide‐diameter supports by thickening their stems and by reducing their twining curvatures. This insight helps us better understand climbing processes and dynamics of stem‐twiners in forest communities and ecosystems.

## 1 INTRODUCTION

Climbing habits are well developed in climbers as strategies to compete with self‐supporting plants for light, space, and survival opportunity (Gentry, 1991; Paul & Yavitt, 2011). The abundance of climbing plants in both extant (Gentry, 1991; Hu & Li, 2015) and fossil (Burnham & Santanna, 2015) records implies that climbing is a successful competitive strategy with a long evolutionary history. Climbing plants account for nearly one‐tenth of the global spermatophyte flora and up to 20%–30% of tropical forest flora (Gentry & Dodson, 1987; Hu & Li, 2015; Jongkind & Hawthorne, 2005; Paul & Yavitt, 2011). They are generally classified into four major categories: tendril‐climbers, root‐climbers, scramblers, and twiners (Hu, Li, & Li, 2010; Paul & Yavitt, 2011; Putz & Holbrook, 1991). Twiners are the largest subdivision, and the majority of them are stem‐twiners, such as morning glories and honeysuckle (Darwin, 1865; Hu et al., 2010; Putz & Holbrook, 1991). Seedlings of stem‐twiners usually grow upright in their juvenile stage, and then the growing tip spirals in a circular motion, known as circumnutation, until it finds a suitable support in its vicinity, usually a stem or a branch of a plant. Once attached, the shoot grows in a continuous, helical trajectory around the support (Goriely & Neukrich, 2006; Paul & Yavitt, 2011; Putz & Holbrook, 1991). This fascinating growth form has attracted the interests of naturalists since Darwin's time. Darwin's insights intrigued many researchers, eventually leading to revelations on the regularity, stability, and mechanics of the helical structure formed by stem‐twiners (Goriely & Neukrich, 2006; Hendricks, 1919; Isnard, Cobb, Holbrook, Zwieniecki, & Dumais, 2009). Usually, a twining shoot will encounter a variety of potential supports during its ascension on a host plant, some of which will prove too weak to support the weight of the shoot while others will be too thick for the shoot to develop stable structure (Darwin, 1865; Peñalosa, 1984). Therefore, insight into climbing capabilities and ascending efficiencies of twining shoots are important for understanding the climbing process of stem‐twiners in forests.

The success rate on thick supports is the most important parameter associated with climbing capacity, whereas the curvature of the helical trajectory is the most important indicator of the ascending efficiency of a stem‐twiner. Experimental studies have implied that herbaceous twining shoots will form stable helixes with almost constant curvature on cylindrical supports and that the curvature will also remain constant on cylindrical supports of different diameters (Bell, 1958; Hu & Li, 2014; Putz & Holbrook, 1991). This relationship is known as the constant curvature hypothesis, and it has been often‐accepted in recent studies (Bastien & Meroz, 2016; Gianoli, 2015; Goriely & Neukrich, 2006). However, only three single‐species case studies, based on small sample sizes, have directly supported this hypothesis, namely studies on *Humulus lupulus* (Bell, 1958), *Dioscorea bulbifera* (Putz & Holbrook, 1991) and *Mikania micrantha* (Hu & Li, 2014). Geometrically, the ascent angle of shoots twining with constant curvature decline dramatically with increasing support thickness, and so their success rate and ascending efficiency decline as well (Hu & Li, 2014). It is intriguing how stem‐twiners overcome the negative effects of constant curvature as they ascend. In addition, studies of *D. bulbifera* and *M. micrantha* have detected that curvature is affected by the diameter of the twining stem (Hu & Li, 2014; Putz & Holbrook, 1991). It is reasonable to expect that stem curvature may be affected by other factors as well. Many environmental factors, such as light (Carter & Teramura, 1988), temperature (Hu et al., 2010), water (Schnitzer, 2005), and support incline (Tao & Zhong, 2003; Zhao, Yu, et al., 2009), have been reported to affect the growth or abundance of twiners or other types of climbers. However, whether and how these factors affect twining behavior or ascending efficiency have not yet been studied in stem‐twiners.

In this study, we conducted a series of experiments with five herbaceous stem‐twining plants (*Ipomoea triloba*,* I. nil*,* Phaseolus vulgaris*,* Vigna unguiculata,* and *M. micrantha*) to test the constant curvature hypothesis. We investigated the effects of supports (support thickness, inclination) and environmental factors (temperature, illumination) on success rate and internode parameters (curvature, diameter, and length). Comprehensive effects of external factors on the behaviors of stem‐twiners are also discussed.

## 2 MATERIALS AND METHODS

Five different species of herbaceous stem‐twiners (viz. *I. triloba* [Convolvulaceae], *I. nil* [Convolvulaceae]*, M. micrantha* [Compositae]*, P. vulgaris* [Leguminosae], and *V. unguiculata* [Leguminosae]) without any additional climbing strategy were selected for this study. In the wild (specifically in the Pearl River Delta where our experiments were conducted), twining stems of these species rarely grow more than 3 mm in diameter. The branching pattern of *V. unguiculata* and *P. vulgaris* is typically sympodial (i.e., when the growth of an apical bud is terminated, its growth continues by a lateral bud and thus the growth direction [ascent angle] is modified as well). The growth of almost all of the internodes of *P. vulgaris* and *V. unguiculata* was modified. A similar phenomenon rarely occurs in *Ipomoea* species, and the branching pattern of *M. micrantha* is typically monopodial.

Seeds of *I. triloba*,* I. nil,* and *M. micrantha* were collected from healthy, wild plant populations in Zhuhai, China (113°35′E, 22°21′N), while seeds of *P. vulgaris* and *V. unguiculata* were collected from cultivated populations in Guangzhou, China (113°17′E, 23°06′N). All experiments were conducted under adequate soil‐nutrient and moisture conditions in Guangzhou during the 2013–2016 period. The experimental site was covered with a plastic film roof about 5 m in height (to prevent the impact of rain) and surrounded by wire mesh (to prevent animal interference).

**Figure 1:**
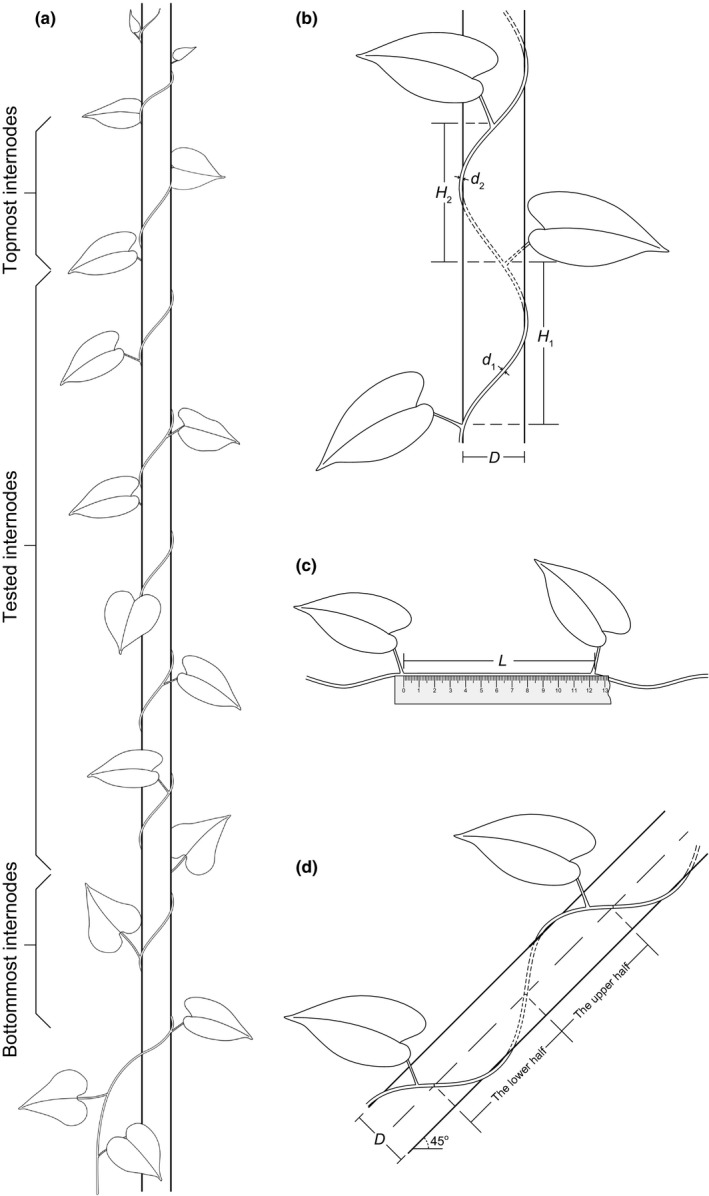
Illustrations of twining stems on vertically erect and 45° inclined cylindrical poles with diameter of *D*. (a) All internodes that contacted the support pole tightly were tested except for the two bottommost and the two topmost internodes. The two bottommost internodes were excluded because they were influenced by the initial contact angle between the shoot and the support, and the two topmost internodes were excluded because they were still twining and elongating. (b) The location of each node was pinpointed and marked, and the height (*H*) and stem diameter (*d*) of each internode were measured with vernier caliper. (c) The length (*L*) of each internode was measured when the twining stems were removed from their supports. (d) For plants grown on 45° inclined poles, each helical circumference was divided into two semicycles (the upper half and the lower half). Each cross point of twining stem and middle lines (both sides) of the pole was pinpointed and marked. The height and length of each semicycle were measured

Four species (*I. triloba*,* I. nil*,* P. vulgaris,* and *V. unguiculata*) were selected to test the effect of support thickness (*D*) on internode curvature (β). For all tested species, seedlings were growth from seeds in humus‐rich soil, and only vigorous plants with sturdy stems were selected and thinned for the experiments. Each plant was supplied with a uniform cylindrical PVC pole (1.8 m in height, wrapped with abrasive paper to simulate coarse bark) and leaned gently against the pole to ensure that its shoot could find and climb the pole. All active axillary buds of each shoot were carefully cut off before they started to twine to ensure that only one stem twined each pole. The *D* levels were designed based on success rates in pilot experiments with small samples. Three different pole sizes (*D *=* *10, 21, and 33 mm) were supplied for the two leguminous plants (*P. vulgaris* and *V. unguiculata*), and four different pole sizes (*D *=* *10, 21, 33, and 41 mm) were supplied for the two *Ipomoea* species. For each species, all treatments were carried out at the same time and under the same soil and water conditions. For each twining stem that grew to 1.5 m in height on its support pole, we measured the height (*H*), length (*L*), and diameter (*d*) of all internodes that contacted the support pole tightly, except for the two bottommost and the two topmost internodes (Figure 1a). The β was calculated as: β =* *2(*L* ^2^ − *H* ^2^)/(*L* ^2^ *D*). Ascent angle (α) was calculated as α = arcsin (*H*/*L*). The success rate (SR) of each *D* level was calculated as the ratio of the number of shoots successfully grew to 1.5 m in height to the number of tested shoots.

Based on the results from the above‐described experiments, we then investigated the effects of three other factors. (1) To test the effect of temperature (*T*, mean temperature during climbing growth period) on internode curvature, *I*.* triloba*,* I. nil*,* P. vulgaris,* and *M. micrantha* plants grown on 21‐mm‐diameter poles at different times of the year were compared. We replaced *V. unguiculata* with *M. micrantha* in this experiment due to the extremely low‐success rate of *V. unguiculata* in previous experiments. Experiments on two *Ipomoea* species were carried out first and their growth under three *T* conditions were compared. Based on the results of *Ipomoea* species, only two *T* conditions were tested using *P. vulgaris* and *M. micrantha* (The temperature difference between the two conditions was more than 5°C). (2) To examine the effect of illumination on internode curvature, we compared *I. triloba* and *I. nil* plants climbing erect, 21‐mm‐diameter poles in full sunlight versus under 50% shade (achieved with layers of shading film). (3) To test the effect of support incline on twining curvature*,* we compared *I. triloba* plants grown on erect (90°) versus inclined (45°) 21‐mm‐diameter poles. Due to space limitations, the inclination experiments were performed in two batches. That is, the poles were inclined eastward or westward in the first experimental batch and southward or northward in the second batch. Each batch had its own independent control group (erect poles). For each shoot that grew to 1.5 m along the inclined pole, we divided each helical circumference into two semicycles: the upper half and the lower half (Figure 1d). The curvature of each semicycle was calculated, respectively.

Data were calculated and analyzed with IBM SPSS Statistics for Windows (version 21.0, IBM Corp. 2012). Statistical analysis was carried out using a one‐way ANOVA. The partial correlation method was employed to determine the degree of association between curvature and other variables.

## 3 RESULTS

### 3.1 Effect of support thickness (*D*) on stem‐twiners

This study revealed that the success rate (SR) of stem‐twiners decreased with increasing support thickness, and the four tested species differed in their climbing capacity on supports of the same size (Table 1). *Ipomoea nil* shoots had the strongest climbing capacity, followed by *P. vulgaris* and *I. triloba*. All of the tested shoots of *I. nil* successfully ascended supports no more than 33 mm in diameter, and the SR was 80% on the 41‐mm‐diameter supports. All of the tested shoots of *P. vulgaris* and *I. triloba* also successfully ascended the 10‐mm‐diameter supports. Their SRs were lower on larger support diameters and *I. triloba’*s SR declined more significantly. Although *V. unguiculata* had the thickest stems, its SRs were the lowest among all three pole diameters tested (Table 1). Approximately one‐third of *V. unguiculata* stems successfully ascended the 10‐mm‐diameter support poles, while only one of the 32 tested stems successfully ascended the 33‐mm‐diameter support poles. Therefore, *V. unguiculata* will not be discussed further because it had a limited number of internode samples to test.

**Table 1:** Internode characteristics (mean ± *SD*) and success rate (SR) of four herbaceous stem‐twiners grown on erect support poles of different diameters (*D*) in full sunlight

Species	*D* (mm)	Tested shoots	SR (%)	Internode parameter				
				*N*	Curvature (cm^−1^)	Diameter (mm)	Length (cm)	Ascent angle (°)
*Ipomoea triloba*	10	27	100	303	0.29 ± 0.07^a^	1.17 ± 0.22^a^	9.82 ± 1.63^a^	67.6 ± 2.9^a^
	21	23	82.6	282	0.23 ± 0.05^b^	1.32 ± 0.22^b^	9.05 ± 1.28^b^	60.5 ± 4.0^b^
	33	15	66.7	105	0.17 ± 0.04^c^	1.60 ± 0.17^c^	9.69 ± 1.65^a^	58.5 ± 4.8^c^
	41	28	42.9	128	0.16 ± 0.04^d^	1.65 ± 0.25^d^	9.93 ± 1.78^a^	54.9 ± 5.3^d^
*Ipomoea nil*	10	9	100	68	0.25 ± 0.04^a^	1.32 ± 0.14^a^	16.05 ± 1.93^a^	69.4 ± 1.9^a^
	21	11	100	101	0.22 ± 0.03^b^	1.44 ± 0.18^b^	14.00 ± 2.01^b^	61.6 ± 2.5^b^
	33	9	100	89	0.22 ± 0.03^b^	1.46 ± 0.16^b^	12.47 ± 1.76^c^	52.9 ± 2.8^c^
	41	15	80.0	121	0.20 ± 0.04^c^	1.91 ± 0.22^c^	12.83 ± 2.01^c^	50.8 ± 5.0^d^
*Phaseolus vulgaris*	10	20	100	76	0.29 ± 0.08^a^	1.27 ± 0.13^a^	24.02 ± 4.89^a^	67.7 ± 3.2^a^
	21	25	92.0	107	0.28 ± 0.07^a^	1.44 ± 0.32^b^	23.30 ± 5.17^a^	57.6 ± 4.6^b^
	33	27	81.5	100	0.24 ± 0.04^b^	1.61 ± 0.30^c^	23.69 ± 5.11^a^	51.5 ± 4.0^c^
*Vigna unguiculata*	10	79	34.2	73	0.14 ± 0.05^a^	1.91 ± 0.30^a^	31.94 ± 4.76^a^	75.1 ± 3.3^a^
	21	53	22.6	37	0.13 ± 0.04^a^	1.79 ± 0.36^a^	26.79 ± 2.71^b^	68.3 ± 2.8^b^
	33	32	3.1	4	0.14 ± 0.01^a^	1.73 ± 0.05^a^	26.05 ± 1.62^b^	62.0 ± 1.8^c^

*N*, number of samples.

Significant differences (*p *<* *.05) are between groups with different superscripts.

The internode curvature (β) of the tested stem‐twiners was significantly affected by both internode diameter (*d*) and support thickness. (1) The curvature of a twining stem on uniform cylindrical supports was not constant and varied among internodes. For all three species, the internode curvature declined and the internode diameter increased with increasing support thickness. The partial correlation coefficients between internode curvature and internode diameter were both negative and significant for *I. triloba* (*r*_β*d.D*_ = −.342, *df* = 815, *p *<* *.01) and *I. nil* (*r*_β*d.D*_ = −.262, *df* = 376, *p *<* *.05), suggesting that an increase in internode diameter would induced *Ipomoea* species to decrease their twining curvatures. However, no significant correlation was detected between internode curvature and internode diameter for *P. vulgaris* (*r*_β*d.D*_ = −.091, *df* = 280, *p *>* *.05). (2) The partial correlation coefficients between internode curvature and support thickness were all negative and significant for *I. triloba* (*r*_β*D.d*_ = −.415, *df* = 815, *p *<* *.01), *I. nil* (*r*_β*D.d*_ = −.140, *df* = 376, *p *<* *.01), and *P. vulgaris* (*r*_β*D.d*_ = −.271, *df* = 280, *p *<* *.01), suggesting that internode curvatures of herbaceous stem‐twiners are significantly affected by support pole diameter. With wider support pole diameters, internode length was generally longer for *I. nil*. However, there was no similar trend or difference in internode length in response to support thickness for either *I. triloba* or *P. vulgaris*.

The actual ascent angles (α) of twining shoots on thicker supports were significantly larger than the angles predicted by the constant curvature hypothesis. Taking *I. triloba* as an example, if twining shoots maintained a constant curvature of 0.29 cm^−1^, as they did on the 10‐mm‐diameter support pole, then predicted α values should decline from 67.6° on the 10‐mm‐diameter support pole to 56.5°, 46.2°, and 39.6° on the 21‐, 33‐, and 41‐mm‐diameter support poles, respectively. However, the actual ascent angles for the three supports poles were 60.5°, 58.5°, and 54.9°, obviously steeper than the values predicted using the constant curvature hypothesis. Therefore, a reduction in twining curvature on thick supports increased the climbing success rate and ascending efficiency of shoots climbing those supports.

### 3.2 Effect of temperature (*T*) on stem‐twiners

The success rate of all tested species, except for *M. micrantha,* was not obviously reduced under cool temperature conditions (Table 2). *Ipomoea nil* shoots had the strongest climbing capacity and none of its twining stems slid down their supports in any of the three temperature conditions. Interestingly, the success rate of *I. triloba* increased stably with declining temperature.

**Table 2:** Internode characteristics (mean ± *SD*) and success rate (SR) of four herbaceous stem‐twiners grown on 21‐mm‐diameter, erect support poles in full sunlight under various temperature conditions

Species	Temperature (°C)	SR (%)	Internode parameters			
			*N*	Curvature (cm^−1^)	Diameter (mm)	Length (cm)
*Ipomoea triloba*	27.3	82.6	282	0.23 ± 0.06^a^	1.32 ± 0.22^a^	9.05 ± 1.28^a^
	25.1	90.1	242	0.22 ± 0.06^a^	1.21 ± 0.22^b^	9.56 ± 1.26^b^
	19.2	95.2	206	0.32 ± 0.06^b^	0.93 ± 0.14^c^	11.53 ± 1.49^c^
*Ipomoea nil*	28.4	100	53	0.20 ± 0.03^a^	1.50 ± 0.15^a^	14.74 ± 1.68^a^
	25.0	100	124	0.20 ± 0.03^a^	1.52 ± 0.15^a^	16.01 ± 2.04^b^
	19.5	100	70	0.24 ± 0.05^b^	1.49 ± 0.14^a^	15.92 ± 2.71^ab^
*Phaseolus vulgaris*	28.0	100	40	0.24 ± 0.07^a^	1.65 ± 0.32^a^	17.80 ± 4.35^a^
	22.9	92.3	57	0.29 ± 0.06^b^	1.36 ± 0.12^b^	22.86 ± 4.30^b^
*Mikania micrantha*	26.7	93.8	99	0.14 ± 0.07^a^	2.44 ± 0.45^a^	10.68 ± 1.83^a^
	19.3	80.0	73	0.17 ± 0.06^b^	2.06 ± 0.21^b^	9.58 ± 2.36^b^

*N*, number of samples.

Significant differences (*p *<* *.05) were between groups with different superscripts.

The internode curvature of the tested herbaceous stem‐twiners was remarkably higher at the cool temperature conditions. (1) The results of the two *Ipomoea* species tested showed that minor differences in temperature did not cause any significant variation in internode curvatures, whereas internode curvatures were significantly higher when temperature was more than 5°C lower (Table 2). The results of the previously described support thickness experiments showed that variations in internode curvatures were due in part to differences in internode diameters. In the temperature experiments, the internode diameter of *I. triloba* shoots was significantly less under the cool temperature condition, while the internode diameter of *I. nil* was not different (Table 2). However, partial correlation coefficients between β and *T* were both negative and significant for *I. triloba* (*r*_β*T.d*_ = −.270, *df* = 727, *p *<* *.01) and *I. nil* (*r*_β*T.d*_ = −.398, *df* = 244, *p *<* *.01), suggesting that lower temperatures are related to higher internode curvature in the two *Ipomoea* species we tested. (2) For the tested *P. vulgaris* and *M. micrantha* shoots, their internode curvatures were significantly higher and internode diameters significantly lower when temperature was more than 5°C lower (Table 2). (3) The variation in internode length, however, was not consistent among the species examined. The internode length of *I. triloba* and *P. vulgaris* was longer, while the internode length of *M. micrantha* was markedly shorter under the cooler temperature condition. There was no significant trend for *I. nil* between internode length and temperature.

### 3.3 Effect of illumination (*I*) on stem‐twiners

The climbing capacities of *Ipomoea* species declined significantly under shading stress. (1) Success rate was only 66.7% for *I. triloba* and 50% for *I. nil* under shading stress, significantly lower than in full sunlight (Table 3). (2) Both internode diameter and length of these two *Ipomoea* species significantly decreased under shading stress (Table 3). (3) The elongation rate was slower for *I. triloba* and *I. nil* shoots grown under the shaded condition than it was for shoots grown in full sunlight. On average, it took 63 days for *I. triloba* shoots to grow 1.5 m upward under shading stress, whereas the shoots grown in full sunlight reached this height in 33 days. For *I. nil*, the rates were 40 days versus 22 days. (4) The partial correlation coefficients between internode curvature and illumination were all negative and significant for *I. triloba* (*r*_β*I.d*_ = −.172, *df* = 163, *p *<* *.05) and *I. nil* (*r*_β*I.d*_ = −.327, *df* = 108, *p *<* *.01), indicating that ascending efficiency of *Ipomoea* vines is significantly reduced by shading stress.

**Table 3:** Internode characteristics (mean ± *SD*) and success rates (SR) of two *Ipomoea* stem‐twiners on vertical 21‐mm‐diameter poles

Species	Illumination (%)	Temperature (°C)	SR (%)	Internode parameter			
				*N*	Curvature (cm^−1^)	Diameter (mm)	Length (cm)
*Ipomoea triloba*	100	18.9	92.9	72	0.30 ± 0.05^a^	0.92 ± 0.15^a^	12.03 ± 1.51^a^
	50	17.3	66.7	94	0.32 ± 0.09^a^	0.75 ± 0.10^b^	11.33 ± 1.62^b^
*Ipomoea nil*	100	28.1	100	62	0.20 ± 0.03^a^	1.51 ± 0.15^a^	14.73 ± 1.73^a^
	50	27.6	50.0	49	0.27 ± 0.04^b^	1.01 ± 0.13^b^	12.06 ± 1.95^b^

*N*, number of samples.

Significant differences (*p *<* *.05) were found between groups with different superscripts.

### 3.4 Effects of support pole incline on *Ipomoea triloba*

The movement of *I. triloba* on inclined supports had changed significantly. (1) In the first batch of inclination experiments, where the supports were inclined eastward or westward, the curvatures of the upper semi‐cycles on inclined supports were not significantly different from curvatures exhibited on vertically erect poles, whereas the curvatures of the lower semicycles were significantly higher than those of the upper semicycles (Table 4). More specifically, the curvatures of the lower semicycles increased by 45.8% on eastward‐inclined supports and 40% on westward‐inclined supports compared with curvatures on upper semicycles. However, no significant curvature differences were found between the two different incline directions. (2) The results of the second batch of inclination experiments were similar to the first batch. However, all curvatures increased as a result of the decrease in experimental temperatures. The curvatures of the lower semicycles were 45% more on the southward‐inclined support and 72% more on the northward‐inclined support in comparison with curvatures on the upper semicycles. (3) Regarding vine growth upward along supports, it took 23–59 days (mean, 36 days) for the *I. triloba* shoots grown on 45° inclined supports to reach 1.5 m elongation along the poles, whereas plants grown on erect supports took 23–49 days (mean, 33 days). It is interesting that the leaves that grew from the lower semicycle, by twisting their petioles, were all arranged along the sunward sides of the support to avoid shading by the support pole.

**Table 4:** Curvature (mean ± *SD*) of *Ipomoea triloba* shoots on erect and 45° inclined support poles (21‐mm‐diameter) in full sunlight

Temperature (°C)	Treatment	Incline direction	Semicycle	*N*	Curvature (cm^−1^)
25.1	Erect	—	—	242	0.22 ± 0.06^a^
	45° incline	Eastward	Upper	59	0.24 ± 0.09^a^
			Lower	63	0.35 ± 0.12^b^
		Westward	Upper	58	0.25 ± 0.10^a^
			Lower	64	0.35 ± 0.10^b^
19.2	Erect	—	—	186	0.31 ± 0.06^c^
	45° incline	Southward	Upper	120	0.31 ± 0.10^c^
			Lower	109	0.45 ± 0.17^d^
		Northward	Upper	119	0.29 ± 0.09^c^
			Lower	120	0.50 ± 0.14^d^

*N*, number of samples.

Significant differences (*p *<* *.05) were found between groups with different superscripts.

## 4 DISCUSSION

### 4.1 Curvature variation of stem‐twiners

Our results revealed that the twining curvature of a certain herbaceous stem‐twiner will vary among internodes and adjust with support thickness. Stems twining on thicker supports would become structurally unstable if they maintained an unvarying curvature as they climbed on slender supports, because constant curvatures elicit a striking decline in ascent angle. Curiously, none of the three, previously published case studies (Bell, 1958; Hu & Li, 2014; Putz & Holbrook, 1991) detected any significant correlation between curvature and support thickness. We believe that perhaps the following three observations could explain this discrepancy. (1) the stem of *H. lupulus*, which was chosen for Bell's experiment, is covered with stiff, deflexed hairs which aid in climbing (Darwin, 1865), and thus may also affect twining curvature values. (2) Although both Putz and Holbrook (1991) and Hu and Li (2014) had detected an effect of stem diameter on curvature, they did not realize that the movement of each separate internode is independent of the others (Darwin, 1865) and that both the diameter and curvature of twining stems is not constant, but changes from one internode to the next. Therefore, methods that sample just one internode from each twining stem (e.g., Hu & Li, 2014), or treat the whole stem as a helix of uniform thickness (e.g., Bell, 1958), cannot accurately demonstrate variations in stem curvatures. (3) None of the previous studies had taken into account the possible impacts of environmental factors on their experiments. That is, the experimental conditions of their different treatments were not described, and thus, it is unknown whether results of their different treatments could be compared.

All results in the present and two previous studies (Hu & Li, 2014; Putz & Holbrook, 1991) have suggested that the curvature of herbaceous twining shoots decrease with increasing stem (internode) diameter. The final stability of a herbaceous twining stem is due, in part, to the development of secondary lignified tissues, which increases with its increasing stem diameter (Hendricks, 1919, 1923). However, stem diameter is not the only parameter determining the tenacity of a twining stem. For instance, the degree of lignification of stem‐twiners with the same stem diameter can dramatically differ among species (Hu, 2009). Thus, the above conclusion (that curvature of herbaceous twining shoots decrease with increasing stem diameter) is applicable to comparisons made between stems from the same species but is not suitable for comparing differences among species.

Our conclusion on curvature variation may apply to stem‐twiners with somewhat woody stems, not just herbaceous ones. The twining movement of a stem‐twiner continues as long as the plant continues to grow, but each separate internode ceases to move as it ages (Darwin, 1865). This situation has also been found on woody stem‐twiners twining with their young herbaceous shoots or stem‐twiners with limited radial thickening capacity, such as *Periploca graeca* (Palm, 1827) and *Merremia boisiana* (Li, Cheng, Liu, & Yu, 2006). Therefore, twining behaviors only exist transitorily in the uppermost several internodes of a climbing shoot (Darwin, 1865). Twining behaviors of these fresh internodes will cease in a few days, although the stem may keep thickening. Therefore, the upper limit of the support thickness is mainly determined by the curvature formed in the uppermost part of the twining shoot (Darwin, 1865; Li et al., 2006). As a result, most stem‐twiners are adapted to ascend supports of moderate thicknesses, and so the relative abundance of stem‐twiners may decrease as support thicknesses increase (Darwin, 1865; Gianoli, 2015; Yuan, Liu, Yang, & Li, 2010).

### 4.2 Effects of external supports on stem‐twiners

The physiology and behavior of twining plants are significantly affected by external supports and their structures (Fan, Wang, & Liu, 2015; Scher, Holbrook, & Silk, 2001; Yan, Qi, Liu, & Zeng, 2007). On the one hand, support availability will affect internode morphology, biomass allocation patterns, and foraging strategies (Gianoli, 2002; Peñalosa, 1984; Yang et al., 2014). Shoots without support will allocate more energy in their early stages to growing stems in their growth toward supports or generate more lateral branches to raise their chances of encountering suitable supports (den Dubbelden & Oosterbeek, 1995; Peñalosa, 1984). In addition, internode diameters of twining shoots on support poles are significantly thicker than stems of shoots without supports (Gianoli, 2002). On the other hand, support thickness affects the twining force and stability of climbing stems (Scher et al., 2001). Stems twining on thicker supports are always thicker than stems twining on slender supports, a situation that is applicable to woody stem‐twiners as well (Yan et al., 2007). It seems that the increase in twining stem diameter on thicker supports is entirely the result of passive selection because weak shoots cannot successfully ascend thicker supports (Hu & Li, 2014). In the present study, however, we detected that the internode diameter of *I. nil* vines increased steadily with increasing support thickness on supports no more than 33 mm in diameter, without any of the tested shoots slipping down the support. This implies that there is another possibility—stems growing on the slender supports do not need to be thick because they have greater twining loads (normal force per unit stem length) than do plants on thicker supports and that their cylindrical helical structures are very stable (Scher et al., 2001). In contrast, stems twining on thicker supports may be structurally unstable and so they have to grow thicker (or more lignified) to develop the extra forces they need to resist gravity or the extreme forces generated by the winding of their uppermost internodes (Rowe, Isnard, Gallenmüller, & Speck, 2006; Scher et al., 2001). In fact, the twining force may increase with developmental stage after cessation of twining and primary growth (Scher et al., 2001). Therefore, species with a higher potential for radial thickening could be more pre‐adapted to climbing thicker supports, and thus, the increase in stem diameter on thicker support poles may be partially attributable to the positive adaptive response of their shoots to climbing thick supports.

Twining plants always encounter inclined branches during their ascent up a host plant. The inclined direction of branches, according to our results, does not affect the climbing behavior or growth rate of twining plants. However, the apical buds of *I. nil* shoots on an inclined support tend to bend upward quickly from lower semicycle positions (with small ascent angles), resulting in a larger curvature on the lower semicycle. The support inclination has also been found to affect the growth of the tendril‐climber, *Trichosanthes kirilowii* (Tao & Zhong, 2003), and the stem‐twiner, *Ipomoea cairica* (Zhao, 2008). Tao and Zhong attributed this influence on growth to self‐shading. However, self‐shading cannot explain the twining behavior of a single *I. triloba* stem on an inclined support pole. We speculate that the difference in twining behavior between the upper and lower semicycles of *I. triloba* vines may be mainly due to phototropism and negative gravitropism. Phototropism, which was observed by Darwin in many stem‐twiners, may induce twining shoots to bend toward light, so that their movement is often accelerated or retarded in traveling to or from the light (Darwin, 1865). Gravity provides an almost constant stimulus and provides important clues for orientating plant growth (Hangarter, 1997; Vandenbrink, Kiss, Herranz, & Medina, 2014). If a twining shoot is accidently forced into an inclined position (e.g., its support falls over), it soon bends upwards, by deviating from its original direction of growth (Darwin, 1865).

### 4.3 Effects of environmental factors on stem‐twiners

The seedlings of stem‐twiners are abundant in high‐light environments where small‐diameter supports are common (Paul & Yavitt, 2011). Their twining shoots may twist together to avoid slipping from their host (Hu & Li, 2014; Putz & Holbrook, 1991). In a closed forest, however, the density of stem‐twiner shoots is usually low in the understory, and they have to generate solitary creeping, nontwining stolons in a random direction to grow toward suitable supports (Liu & Wang, 2009; Peñalosa, 1984). When a stolon encounters a suitable support, which triggers twining behavior, the twining shoot sprouts and tries to ascend into the forest canopy again (den Dubbelden & Oosterbeek, 1995; Peñalosa, 1984). Thus, the climbing capacity of a single shoot under shading stress is an important factor restricting its clonal expansion capacity in a closed forest. On the one hand, the stems of herbaceous stem‐twiners are always thinner under shaded conditions (Gianoli, 2003); on the other hand, both climbing and searching for light typically involve stem elongation at the cost of leaf surface area (Valladares, Gianoli, & Saldana, 2011). We did not evaluate changes in biomass allocation under shading stress, but both the internode diameter and internode length of the two *Ipomoea* species we studied were less under shade; likewise, the ascending efficiency of their stems was significantly less in shade.

The effect of low‐temperature stress on twining curvature is similar to that of shading stress. Since both light and temperature can affect the curvature of *Ipomoea* vines, it is reasonable to infer that fluctuations in their curvature patterns can be partially attributed to diurnal changes in amount of illumination and temperature. Experiments on *P. vulgaris* suggested that photoperiods of 12 versus 18 hr did not affect the time during which climbing occurred or the success rate (Kretchmer, Ozbun, Kaplan, Laing, & Wallace, 1977), but the effect of day length on curvature was unclear. Darwin (1865) reported that the revolution rate of a twining stem was nearly the same during the night and the day in all the plants he observed; and he inferred that the circadian rhythm may retarding one semicircle and accelerating the other, so as not to modify greatly the rate of the whole revolution. However, it is difficult to accurately quantify the impact of circadian rhythm on the final curvature of any particular internode because there are always two or more internodes winding and elongating concurrently as a twining shoot grows up its host (Darwin, 1865; Isnard & Silk, 2009). We tracked the elongation of several internodes of *I. triloba* and *I. nil* in a separate experiment carried out in midsummer, and the internode elongation rates of *I. triloba* and *I. nil* during the day were 2.4 times and 1.4 times of that at night (unpublished data). Additional research that focuses on the impact of diurnal changes on the movement of twining plants is urgently needed.

Other environmental factors, including water and soil conditions, may also affect the behavior and efficiency of herbaceous stem‐twiners (Atala, Cordero, & Gianoli, 2011; Liu & Duan, 2013). Herbaceous stem‐twiners, like other types of climbers, are more efficient in conducting water than trees because their vessel diameters are often greater than vessel diameters in closely related species of self‐supporting plants (Ewers & Fisher, 1991; Paul & Yavitt, 2011). However, relatively larger vessels may mean that they are less tolerant to drought stress because the stems of herbaceous plants, especially annuals, are supported by turgor pressure (Struik, 1965). Thus, drought stress may reduce stem rigidity and interfere with a stem‐twiners’ ability to maintain tight contact with its support. The growth and movement of twining plants have also been confirmed to be affected by soil pH (Hu, Deng, Zhang, & Li, 2016; Wu, Xing, Zhu, & Liang, 2009; Zhao, Li, et al., 2009), ions (Millet & Badot, 1996) and nutrient levels (Hou, Peng, Chen, & Ni, 2011). For instance, the bending zone displays higher K^+^ and malate concentrations than the upper and lower parts of shoots (Millet & Badot, 1996). However, it is unclear how soil conditions could affect twining movement and curvature.

## 5 CONCLUSIONS

Searching for suitable external supports is very important for the ascent and vegetative spread of stem‐twiners. It is also important for understanding the ecological processes and dynamics of stem‐twiners in forests. However, our knowledge of stem‐twiners is extremely inconsistent with its diversity. Furthermore, most climber‐related studies have focused on large‐scale dynamic processes associated with growth in the horizontal direction, with little attention given to vertical ascending processes associated with special climbing capabilities. The present study rejects the often‐accepted constant curvature hypothesis and reveals that twining curvature and ascending efficiency of certain stem‐twiners vary among internodes and adjust in response to changes in inclination of external supports and environmental factors (such as temperature and degree of shading). We speculate that herbaceous stem‐twiners adapt to climbing thick supports by thickening their internode diameters and by reducing their twining curvatures, which are both conducive to successful attachment and efficient ascent of its shoots. Our study sheds light on the key processes inherent to the vegetative spread of herbaceous stem‐twiners. We inferred that some of our conclusions may be partially applicable to woody stem‐twiners that have a limited capacity for radial thickening and to large lianas with herbaceous or semi‐woody twining shoots. These conclusions should be tested and verified by further study on climbing behaviors at various stages of woody stem‐twiners. We expect that more research will be devoted to vertical dynamic processes of stem‐twiners and other types of climbers.

## CONFLICT OF INTEREST

None declared.

## AUTHORS CONTRIBUTION

Hu L. conceived and designed the experiments, Hu L., Chen Y.F., and Liu M.C. performed the experiments. Chen Y.F. and Liu M.C. performed the statistical analyses. Hu L. wrote the first version of the manuscript. All authors contributed to the final version of the manuscript.
